# Prediction of Sarcopenia Onset in Older Adults with Diabetes using Urinary Titin Levels: A Japanese Cohort Study

**DOI:** 10.1093/geroni/igaf122.4177

**Published:** 2025-12-31

**Authors:** Michio Shimabukuro, Hayato Tanabe, Kiriko Watanabe, Rie Tsutsumi, Yuna Izumi-Mishima, Kazuhiro Nomura, Masafumi Matsuo, Hiroshi Sakaue

**Affiliations:** Fukushima Medical University School of Medicine, Fukushima, Fukushima, Japan; Department of Diabetes, Endocrinology, and Metabolism, Fukushima, Fukushima, Japan; Fukushima Medical University School of Medicine, Fukushima, Fukushima, Japan; Institute of Biomedical Sciences, Tokushima University, Tokushima, Tokushima, Japan; Institute of Biomedical Sciences, Tokushima University, Tokushima, Tokushima, Japan; Institute of Biomedical Sciences, Tokushima University, Tokushima, Tokushima, Japan; Kobe University, Kobe, Hyogo, Japan; Institute of Biomedical Sciences, Tokushima University, Tokushima, Tokushima, Japan

## Abstract

Standardized biomarkers for incident sarcopenia are still elusive. Recently, we reported that urinary titin serves as a novel, noninvasive biomarker to identify individuals with diabetes at a high risk of muscle damage and sarcopenia (Tanabe, et al. Diabetes Care in press). However, the predictive power of urinary titin for sarcopenia has not yet been clarified in older adults. In Japanese older adults with type 2 diabetes, baseline urinary titin levels were measured, and sarcopenia was evaluated annually using the Asian Working Group for Sarcopenia (AWGS) 2019 criteria. Kaplan-Meier curves, Cox models, and time-dependent receiver operating characteristic analyses were employed to assess incident sarcopenia. Among the 287 participants (median follow-up: 824 days), 39 developed sarcopenia. The high titin tertile was associated with an elevated sarcopenia risk (log-rank P = 0.008) and low muscle strength (P = 0.004). Cox models associated titin with sarcopenia (adjusted hazard ratio per SD: 1.38, 95% CI: 1.07–1.78, P = 0.013) and low muscle strength (HR per SD: 1.37, 95% CI: 1.10–1.70, P = 0.005). Risk estimates were consistent across subgroups, including males, individuals with BMI< 25 kg/m2, HbA1c ≥7%, and eGFR ≥60 mL/min/1.73 m2 (P for interaction >0.05). Elevated urinary titin levels predict sarcopenia and low muscle strength in older adults with type 2 diabetes, supporting its use as a noninvasive biomarker in this population.

